# Aging mitochondria in the context of SARS-CoV-2: exploring interactions and implications

**DOI:** 10.3389/fragi.2024.1442323

**Published:** 2024-09-24

**Authors:** M. Victoria Delpino, Jorge Quarleri

**Affiliations:** Consejo Nacional de Investigaciones Científicas y Tecnológicas (CONICET), Instituto de Investigaciones Biomédicas en Retrovirus y Sida (INBIRS), Laboratorio de Inmunopatología Viral, Universidad de Buenos Aires (UBA), Buenos Aires, Argentina

**Keywords:** COVID-19, SARS-CoV-2, mitochondria, ROS, obesity

## Abstract

The coronavirus disease 2019 (COVID-19), caused by severe acute respiratory syndrome coronavirus 2 (SARS-CoV-2), has presented global challenges with a diverse clinical spectrum, including severe respiratory complications and systemic effects. This review explores the intricate relationship between mitochondrial dysfunction, aging, and obesity in COVID-19. Mitochondria are vital for cellular energy provision and resilience against age-related macromolecule damage accumulation. They manage energy allocation in cells, activating adaptive responses and stress signals such as redox imbalance and innate immunity activation. As organisms age, mitochondrial function diminishes. Aging and obesity, linked to mitochondrial dysfunction, compromise the antiviral response, affecting the release of interferons, and worsening COVID-19 severity. Furthermore, the development of post-acute sequelae of SARS-CoV-2 infection (PASC), also known as long COVID has been associated with altered energy metabolism, and chronic immune dysregulation derived from mitochondrial dysfunction. Understanding the interplay between mitochondria, aging, obesity, and viral infections provides insights into COVID-19 pathogenesis. Targeting mitochondrial health may offer potential therapeutic strategies to mitigate severe outcomes and address long-term consequences in infected individuals.

## Introduction

The coronavirus disease 2019 (COVID-19), resulting from an infection with the severe acute respiratory syndrome coronavirus 2 (SARS-CoV-2), has had a catastrophic impact worldwide in the 21st century. The diverse clinical spectrum, spanning from asymptomatic and mild upper respiratory tract illness to severe interstitial pneumonia with respiratory failure and, in some cases, death, has presented significant challenges in managing the COVID-19 pandemic (Menni et al., 2022; Nyberg et al., 2022).

Primarily, COVID-19 patients experience a respiratory tract infection. Unfortunately, a substantial number of individuals face severe and fatal consequences due to an escalation of inflammatory events (Zhang et al., 2020). This heightened inflammatory state is reportedly linked to detrimental systemic occurrences, such as oxidative stress, disruptions in iron homeostasis, increased blood clotting, and the formation of thrombi (Terpos et al., 2020; Qiu et al., 2024).

Various biomarkers associated with inflammation and thrombosis have been identified as predictors of mortality in critically ill COVID-19 patients. Lymphopenia has been highlighted as a crucial feature and proposed as a potential prognostic marker. Additionally, the elevation of D-dimer and interleukin-6 (IL-6) levels has been observed to escalate with the progression of the disease (Elbadawy et al., 2023; Mahmood et al., 2023), correlated with an increased risk of mortality (Tang et al., 2020; Mahmood et al., 2023). Ferritin, an intracellular protein for iron storage, plays a crucial role in immune dysregulation. It is an acute-phase protein that rises in concentration during various inflammatory diseases. Such elevated levels of ferritin, known as hyperferritinemia, have been linked to increased disease severity and unfavorable clinical outcomes in COVID-19, including a higher risk of mortality and long term outcomes (Hulkoti et al., 2022; Kurian et al., 2023; Hanson et al., 2024). Likewise, transferrin receptor 1 (TfR1) expression is also elevated. It is a specific marker for ferroptosis and regulates iron homeostasis by transporting holo-transferrin, which contributes to the intracellular labile iron pool. The increase in these two iron regulatory proteins is closely associated with iron overload, which promotes cell death via ferroptosis, as a form of non-apoptotic cell death that depends on iron and is driven by phospholipid peroxidation (Qiu et al., 2024). Among others, it is favored by SARS-CoV-2-mediated inhibition of the nuclear factor erythroid 2-related factor 2 (NRF2)-mediated antioxidant response, which normally protects cells from oxidative damage (Qu et al., 2023).

Oxidative stress has been identified as a significant contributor to the pathogenesis and severity of COVID-19 (Delgado-Roche and Mesta, 2020; Laforge et al., 2020). Accordingly, examinations of lung tissue in COVID-19 patients reveal a disruption in the balance of mitochondrial dynamics, giving rise to oxidative stress, a pro-inflammatory state, increased cytokine production, and cell death (Ganji and Reddy, 2020). Dysfunctional mitochondria, with heightened reactive oxygen species (ROS) and decreased ATP production, are associated with aging-related traits such as visceral obesity, insulin resistance, and reduced mobility (Fabbri et al., 2017; Zampino et al., 2020; Zampino et al., 2021; Tian et al., 2022). Besides, mitochondria play a crucial role in supporting the effector responses of the immune system. Mitochondrial Damage-Associated Molecular Patterns (mtDAMPs) emerge as potent immunological activators discharged from damaged or dying cells into the cytoplasm or circulation, thereby exerting an influence on immunity (Scozzi et al., 2021). These inflammatory signals perpetuate a cycle of events that exacerbate mitochondrial oxidative damage, thereby contributing to significant systemic alterations.

Under circumstances of mitochondrial dysfunction, such as those observed in aging and obesity, these occurrences might be further intensified (Lizzo et al., 2022; Nidadavolu et al., 2023; Kivimaki et al., 2024). Additionally, mitochondrial dysfunction could potentially serve as a fundamental mechanism in the development of long COVID. This is attributed to a virally induced persistent metabolic imbalance that fails to resolve, marked by ongoing mitochondrial dysfunction. In this state, ROS persistently fuel inflammation (Dirajlal-Fargo et al., 2024; Martinez et al., 2024).

This review focuses on the current understanding regarding the role of mitochondria in elderly individuals infected with the virus and the role of age-dependent fat accumulation. We will place particular emphasis on exploring the role of mitochondria in the long-term consequences, to provide insights into potential strategies for alleviating the aftermath of the disease.

## The mitochondria

Mitochondria, double-membrane enclosed sub-cellular organelles, are primarily recognized for their function as cellular energy generators through oxidative phosphorylation in eukaryotic cells. Throughout this process, mitochondria extensively utilize a significant portion of the organism’s inhaled oxygen (Osellame et al., 2012). Mitochondria play a crucial role in maintaining cellular and organismal health, as they are the primary contributors to generating the majority of energy required to fuel biological processes in eukaryotes (Casanova et al., 2023).

Mitochondria also serve as potential initiators of inflammation and cell death. This occurs when ROS or mitochondrial DNA (mtDNA) leak from the organelles, activating inflammasomes or cytosolic DNA sensors, respectively, leading to cellular demise. Aging leads to mitochondrial dysfunction through various intertwined mechanisms, including the accumulation of mtDNA mutations, impaired proteostasis destabilizing respiratory chain complexes, decreased organelle turnover, and alterations in mitochondrial dynamics. Consequently, mitochondrial contribution to cellular energy production diminishes, while ROS production increases, potentially causing mitochondrial membrane permeabilization, inflammation, and cell death. Maintaining mitochondrial function is crucial for overall health, and its decline significantly contributes to the aging process (Suomalainen and Nunnari, 2024). It is widely recognized that mitochondria form dynamic organelle networks, undergoing processes of fusion, division, and interactions with other cellular structures. These activities play a pivotal role in orchestrating numerous processes that govern cellular fitness, longevity, and fate (Chen et al., 2023). On the flip side, substantial research indicates that mitochondrial dysfunction plays a role in various metabolic disorders and numerous pathologies, whether infectious or not (Behl et al., 2023).

Mitochondrial function plays a dynamic role in actively influencing the process of normal aging. The connections between mitochondria and the aging process have been acknowledged for quite some time (Short et al., 2005a; Short et al., 2005b; Trifunovic et al., 2005). Production of low ROS levels in mitochondria promotes longevity and connects mitochondrial function and healthy aging (Schulz et al., 2007).

Research into mitochondrial network dynamics has revealed that the state of mitochondrial morphology, whether fused or fragmented, directly impacts the various functions of cellular processes (Youle and van der Bliek, 2012). The dynamics of mitochondria themselves change with age. Investigations using *in vivo* models have demonstrated that the mitochondrial network in aged animals tends to be more heterogeneous, fragmented, and composed of large, swollen mitochondria that resist elimination through mitophagy (Yasuda et al., 2006; Leduc-Gaudet et al., 2015; Brandt et al., 2017; Rana et al., 2017). However, whether they actively contribute to aging by modulating mitochondrial health or are a passive consequence of the aging process deserves to be elucidated.

## Aging and mitochondria

The most severe cases of COVID-19 are identified in elderly male individuals who are also dealing with overweight or obesity (Gholi et al., 2023; Altunkaya et al., 2024; Goto et al., 2024). Two factors, aging and obesity, are closely linked to mitochondrial dysfunction (Chen et al., 2024; Liabeuf et al., 2024; Tolstik et al., 2024; Yang et al., 2024). Mitochondria play a crucial role in various cellular functions, contributing to energy production and processes such as nucleotide synthesis, the regulation of calcium homeostasis, and the modification of membrane phospholipids (Spinelli and Haigis, 2018). Additionally, malfunctioning mitochondria serve as the primary generators of reactive oxygen species (mtROS), leading to heightened oxidative damage in the contexts of aging and metabolic diseases (Zhang et al., 2022).

As individuals age, DNA damage accrues, contributing to age-related phenotypic alterations (Schumacher et al., 2021). mtDNA is significantly affected by aging-related mutations and deletions, attributed to its high replicative rate, inefficient repair mechanisms, oxidative surroundings, and absence of protective histones. Somatic alterations in mtDNA rise across human tissues with age, yet the extent to which this affects the aging process at a functional level remains uncertain (Suomalainen and Nunnari, 2024). These mutations often result in mtDNA deletions, which subsequently impair oxidative phosphorylation (Fontana and Gahlon, 2020). Consequently, defective mitochondria can proliferate clonally alongside functional ones, leading to an increased accumulation of unproductive mitochondria in the cells of older individuals.

Mammalian cell DNA undergoes modifications daily, including ROS-induced single-strand breaks detected by poly (ADP-ribose) polymerase 1 (PARP1), and subsequently repaired by specific mechanisms (Eustermann et al., 2011). However, sustained PARP1 activation leads to NAD + depletion, crucial for sirtuin (SIRT) function, particularly impairing mitochondrial SIRT3 activity and affecting antioxidant systems, mitochondrial DNA repair, quality control, and biogenesis pathways, possibly promoting senescence (Verdin, 2015; Wiley et al., 2016). Declining NAD + levels also hamper SIRT2 activity, which normally inhibits the NLR family pyrin domain-containing 3 (NLRP3) inflammasome, thus suppressing inflammation (He et al., 2020). NAD + supplementation counteracts premature aging manifestations linked to DNA repair deficiencies by restoring mitochondrial function and mitophagy (Cerutti et al., 2014; Fang et al., 2016). The accumulation of damaged mitochondria by an imbalance in mitochondrial dynamics, autophagy, and biogenesis results in a hallmark of aging-related metabolic syndromes, including obesity (Natarajan et al., 2020).

Autophagy is a complex cellular process involving the seclusion of cytoplasmic material within double-membrane vesicles known as autophagosomes. These vesicles later merge with lysosomes, where their contents are broken down (Son et al., 2022). While traditionally associated with maintaining proteostasis, autophagy also plays a crucial role in degrading non-proteinaceous macromolecules like ectopic cytosolic DNA, lipid vesicles, and glycogen. Furthermore, it targets entire organelles, including dysfunctional mitochondria (mitophagy), as well as other organelles through lysophagy, reticulophagy, or pexophagy (Aman et al., 2021). Notably, a decline in autophagy with age represents one of the primary mechanisms behind reduced organelle turnover.

Such defective autophagy and mitophagy, prevalent in chronic diseases and aging, triggers the release of DAMPs and ROS, thereby activating NLRP3 and inflammation associated with mitochondria (Iyer et al., 2013; Ma et al., 2023). Dysfunctional mitochondria release DAMPs, predominantly oxidized mtDNA and cardiolipin, into the cytosol via apoptosis-activated BAK/BAX macropore assemblies. mtDNA contains conserved unmethylated CpG motifs akin to bacterial DNA. Upon entering the cytosol, it triggers an immune response as “non-self” via pattern recognition receptors like TLR9 and NLRs (Oka et al., 2012). Cardiolipin, a phospholipid typically found in the inner mitochondrial membrane crucial for mitochondrial structure and function, promotes mitophagy when extruded from damaged mitochondria (Chu et al., 2013). In severe cases, cardiolipin can induce cytochrome c release, initiating apoptosis (Li et al., 2019). The release of ROS-damaged mtDNA and cardiolipin into the cytosol activates various inflammatory pathways, notably involving the NLRP3 inflammasome, the cGAS/STING DNA-sensing pathway, and NF-κB (Chen et al., 2016). The NLRP3 inflammasome plays a significant role in aging and age-related ailments, including atherosclerosis, metabolic syndrome, type 2 diabetes, and Alzheimer’s disease (Lee et al., 2013; Ising et al., 2019). In concordance, a recent study revealed that inhibiting neutrophil infiltration mediated by the mtDNA-STING-NLRP3/IL-1β axis protects neurons in Alzheimer’s disease (Xia et al., 2024). Autophagy can remove mitochondria-related DAMPs, pro–IL-1β, and ubiquitinated inflammasome complexes, thereby diminishing the inflammatory milieu (Sun et al., 2017). Inhibition of autophagy results in increased cytosolic-free mtDNA and IL-1β production (Shi et al., 2012). Deleting the NLRP3 inflammasome shields mice from age-associated immune activation, CNS transcriptome changes, and astrogliosis, while also enhancing glycemic control and motor performance (Youm et al., 2013). Loss-of-function mutations in the STING gene confer protection against age-related diseases, particularly in smokers with chronic pulmonary conditions, who typically exhibit a proinflammatory state (Hamann et al., 2019). Conversely, rare gain-of-function STING mutations are linked to severe inflammation, particularly in the lungs. These findings emphasize the crucial role of STING in initiating both innate and adaptive immune responses and suggest that abnormal STING activation is implicated (Jeremiah et al., 2014). NF-κB complexes have been identified within mitochondria, close to the inner membrane, suggesting activation of the NF-κB pathway during mitochondrial dysfunction and oxidative stress, although the exact mechanism remains undefined. The canonical NF-κB pathway is activated by endogenous and environmental factors, including diet and air pollution, primarily through stimulation of proinflammatory receptors such as the TNF receptor superfamily and the IL-1 receptor (Bektas et al., 2018). NF-κB activation is evident in various age-related diseases, including sarcopenia, osteoporosis, and neurodegenerative disorders (Cai et al., 2004; Steinman, 2008; Tilstra et al., 2011; Chen et al., 2013; Walker et al., 2022).

In the context of metabolic syndrome, a crucial contributor to the severity of COVID-19, mitochondrial dysfunction plays a central role in the rise of insulin resistance associated with type 2 diabetes (Ajaz et al., 2021). This association is linked to an upsurge in mutations in mtDNA, alterations in ATP levels, the generation of ROS, and an unbalanced turnover of mitochondria (Singh et al., 2020; Ajaz et al., 2021; Burtscher et al., 2021; Kozlov et al., 2021; Nunn et al., 2022). Metabolic inflexibility, stemming from the accumulation of impaired mitochondria in both metabolic syndrome and aging, leads to insufficient disposal of metabolic substrates. This inadequacy affects the metabolism of glucose and free fatty acids, contributing to a cluster of metabolic abnormalities that impact various organs and tissues, including immune cells (Haythorne et al., 2022; Yuan et al., 2022).

Immunosenescence is characterized as the age-dependent decline of the immune system, reflecting its adaptation to deteriorative changes over time (Pawelec, 2007). Regardless of the perspective, whether viewed as deterioration or adaptive response, immunosenescence, like many other physiological dysfunctions in aging, has been linked in part to dysfunctional mitochondria (Liu et al., 2023). In this context, mitochondrial dysfunction has a specific impact on T-helper CD4^+^ lymphocytes, which play a crucial role in regulating the overall immune response. Consequently, this dysfunction leads to increased senescence in various tissues, triggering a cytokine storm. It becomes evident that mitochondrial dysfunction is a significant factor contributing to the proinflammatory profile (Shimada et al., 2012; Zhong et al., 2018). This occurs through the release of inflammatory cytokines, activated by mechanisms such as the NLRP3 inflammasome, which are overactivated in both aging and metabolic diseases (Youm et al., 2013). A notable characteristic of aging is the onset of a persistent, low-grade, and sterile inflammatory state often termed ‘inflammaging’. This state, identified by increased levels of circulating inflammatory biomarkers such as IL-6 and C-reactive protein, is acknowledged as a significant risk factor for heightened morbidity and mortality in the elderly (Wassel et al., 2010; Puzianowska-Kuznicka et al., 2016; Gu et al., 2019; Kalair et al., 2023).

## Role of mitochondria in antiviral response

Dysfunctional mitochondria have a notable impact on the immune system’s ability to combat viral infections (Marchi et al., 2023). The release of type I interferon alpha (IFNα) and beta (IFNβ), and type III interferon lambda (IFNλ) plays a crucial role in the antiviral response, as these cytokines restrict viral replication, enhance antigen presentation, and stimulate antigen-specific T and B cell responses. Despite minimal protein homology with type I IFNs, IFN-λ shares similar functions: both are induced by viral infections and activate the JAK/STAT pathway, leading to antiviral responses and IFN-stimulated gene (ISG) transcription. However, IFN-λ has a distinct role in protecting barrier organs. Epithelial cells preferentially produce IFN-λ, and its specific receptor, IFNLR1, is highly expressed in epithelial cells. Initially, IFN-λ was considered mainly an epithelial cytokine that restricts viral replication at mucosal sites. Recent research shows that IFNLR1 is broadly expressed, with immune cells like neutrophils and dendritic cells also responding to IFN-λ. In many *in vivo* models, IFN-λ modulates immune cell functions, acting not only as an epithelial-specific cytokine but also as a regulator of the inflammatory response at mucosal sites (Zanoni et al., 2017).

The mitochondrial antiviral signaling (MAVS) pathway is actively involved in the expression of IFN-I and III (Mertowska et al., 2023). MAVS (also known as IPS-1, VISA, and Cardif) is situated on the outer membrane of mitochondria, peroxisomes, and the mitochondria-associated membrane in the endoplasmic reticulum. Retinoic acid-inducible gene (RIG-I) and melanoma differentiation-associated factor 5 (MDA5) are highly conserved cytoplasmic pattern recognition receptors that detect viral RNAs during infection, triggering a series of antiviral signaling pathways that result in the production of type I and III interferons and other pro-inflammatory cytokines. These receptors play distinct roles by detecting different groups of viruses and recognizing specific features of viral RNAs. RIG-I and MDA5 are paralogous, sharing the same domain architecture and the downstream adaptor molecule MAVS. Both receptors possess the N-terminal tandem caspase activation recruitment domain, which interacts with MAVS to activate the antiviral signaling pathway. Following binding with RIG-1 and MDA5, MAVS undergoes aggregation, acting as a scaffold to recruit TNF receptor-associated factors (TRAFs). This cascade leads to the phosphorylation of interferon-regulatory factors (IRFs), facilitating their translocation to the nucleus. This, in turn, induces the synthesis of interferons (IFN) (Kawai et al., 2005).

Knocking down MAVS expression through RNA interference has been shown to eliminate the activation of NF-κB and IRF3 in response to viral infection, allowing for viral replication (Seth et al., 2005). The aging process impacts monocytes’ mitochondrial functions, leading to heightened inflammation and compromised cellular functions, including phagocytosis (Pence and Yarbro, 2018). Aging adversely affects both the primary and secondary RIG-I signaling pathways responsible for regulating the expression of IFN-I genes. Consequently, this impairment hinders the effectiveness of the antiviral response (Molony et al., 2017; D'Souza et al., 2021).

Viral infections contribute to an elevation in mtROS, which drive innate immune inflammation, thereby exacerbating viral pathogenesis. Additionally, many viruses induce alterations in mitochondrial activity, diminishing the cells’ capability to initiate IFN-I-dependent antiviral responses (Elesela and Lukacs, 2021). Furthermore, viruses demonstrate heightened replication efficiency in senescent cells with reduced mitochondrial capacity (Kelley et al., 2020), suggesting that the accumulation of senescent cells, characterized by elevated levels of dysfunctional mitochondria during aging and age-related diseases, may contribute to the progression of viral infections (Liu et al., 2023).

Mitochondrial biogenesis generates new mitochondria to maintain cellular homeostasis and, as recently reported, viruses exploit this process to counteract innate antiviral immunity. The nuclear respiratory factor-1 (NRF1) is involved in this process. Thus, inhibition of NRF1-mediated mitochondrial biogenesis worsened virus-induced mitochondrial damage, increased mtDNA release, mtROS production, and activated the innate immune response. This is a novel antiviral mechanism where an NRF1-mediated feedback loop modulates mitochondrial biogenesis to counteract the innate immune response (Zhao et al., 2023).

In the case of COVID-19, the immune system’s antiviral response is compromised by a low release of type I and III IFN accompanied by high expression of proinflammatory cytokines (Quarleri and Delpino, 2021). The disproportionate response may be linked to the accumulation of damaged mitochondria observed in aging and obesity, significantly impacting the antiviral immunological response (Amorim et al., 2022).

ACE2 is an entry receptor for SARS-CoV-2 and is also linked to mtDNA depletion and mitochondrial dysfunction (Zhao et al., 2022). Transcriptome analysis of clinical samples reveals that cardiac, hepatic, and renal impairments associated with COVID-19 are linked to ACE2, inflammatory cytokine storms, and mitochondrial pathways.

Interestingly, the complex interplay between androgens, ACE2 expression, and the susceptibility of men to COVID-19 highlights potential therapeutic avenues. Antiandrogenic drugs reduce ACE2 expression, which may provide insights into the higher COVID-19 susceptibility observed in men and suggest these drugs as potential COVID-19 therapeutics (Samuel et al., 2020). SARS-CoV-2 binding to ACE2 reduces intracellular ACE2, disrupting mitochondrial regulation. Higher ACE2 levels can help restore mitochondrial function. Androgens induce TMPRSS2, which activates viral spike proteins (Bajpai et al., 2019). Both androgen and estrogen receptors are localized in mitochondria, potentially contributing to the increased COVID-19 susceptibility and mortality seen in men.

These insights point to potential medical interventions for addressing COVID-19-induced multiorgan damage (Chang and Wei, 2024). The assembly of the MAVS complex undergoes modulation facilitated by the mitochondrial translocase of the outer membrane 70 (Tom70) (Liu et al., 2010). *In vitro* studies support this hypothesis by demonstrating that the Orf9b protein of SARS-CoV-2, generated from an alternative open reading frame present in the nucleocapsid (N) gene, interacts with Tom 70 and antagonizes type I and III interferons by targeting multiple components of the RIG-I/MDA-5–MAVS signaling pathways (Gao et al., 2021; Han et al., 2021). Furthermore, antibodies targeting Orf9b were detected in the serum of individuals who had recovered from SARS-CoV-2 infections (Jiang et al., 2020). Hence, the Orf9b protein of SARS-CoV-2 might have a pivotal role in the interactions between the coronavirus and its host, potentially influencing the production of IFN-I.

Recently, it has been found that the SARS-CoV-2 Orf3c protein inhibits immune activation mediated by RIG-I and MDA5, along with the interaction with MAVS, resulting in the subsequent suppression of IFN-β induction (Muller et al., 2023). This Orf3c exhibits conservation across *Sarbecovirus* subgenus members, encompassing SARS-CoV-1 and bat-derived coronaviruses. Notably, in line with its dispensable role, the SARS-CoV-2 delta and kappa variants feature premature stop codons in the ORF3c gene, indicating that this reading frame is not crucial for effective *in vivo* viral replication and is likely compensated for by other viral proteins (Muller et al., 2023).

Numerous lines of evidence indicate that the preservation of mitochondrial integrity is essential for the overall host defense against viral infections. The infection by SARS-CoV-2 appears to induce an increase in mitochondrial fusion, resulting in elongation that prevents apoptosis and creates an intracellular environment conducive to virus propagation in infected cells (Shang et al., 2021; Gabanella et al., 2022; Pliss et al., 2022; Shin et al., 2024). Consequently, viral interaction with mitochondria could modulate the immune response and affect its integrity, worsening the progression of the disease.

## Mitochondria dysfunction in long COVID

Several studies have documented mitochondrial dysfunction in individuals with long COVID-19, shedding light on how this condition may sustain the diverse and persistent symptoms associated with the syndrome. Research has identified abnormalities in mitochondrial respiration, bioenergetics, and mitochondria-related gene expression in peripheral blood mononuclear cells (PBMCs) from long COVID patients (De Vitis et al., 2022; Streng et al., 2022; Dirajlal-Fargo et al., 2024). These abnormalities suggest compromised mitochondrial energy production, potentially causing fatigue and muscle weakness.

The pro-survival role of epidermal growth factor receptors (EGFR), necessary for viral entry and disease development, has been reported in several virus infections, including SARS-CoV-2. Interestingly, this EGFR translocates to the outer mitochondrial membrane (OMM), supporting robust virus propagation during the early stage of infection. Thus, SARS-CoV-2 enhances mitochondrial bioenergetic efficiency to facilitate its propagation, increasing the mitochondrial membrane potential (ΔΨm) through the viral RNA-nucleocapsid cluster. This results in elongated mitochondria, increased oxidative phosphorylation (OXPHOS), and elevated ATP production (Shin et al., 2024). Such mitochondrial alterations play a crucial role in maintaining virus persistence during the early stage of infection and offer novel insights into the antiviral potential of EGFR inhibitors as therapeutic agents for COVID-19.

Accordingly, SARS-CoV-2 pathology can be mitigated by catalytically reducing mtROS, as was revealed in studies using the murine model (Guarnieri et al., 2024).

Martinez et al. employed a multiplatform mass spectrometry-based approach to profile the metabolomics and lipidomics of plasma samples, identifying lipid species and metabolites that distinguished long COVID patients from recovered individuals. Other key findings include decreased amino acid metabolism, ceramide plasma levels, and increased tricarboxylic acid (TCA) cycle activity, indicating impaired mitochondrial function (Martinez et al., 2024). Similarly, severe and critical patients exhibited high histidine-rich glycoprotein (HRG) and cholesteryl ester (ChoE) 20:3 but low alpha-ketoglutaric acid levels, alongside disruptions in the TCA cycle, lipid metabolism, amino acid biosynthesis, and coagulation (Sanchez et al., 2023). These changes may indicate direct viral effects on mitochondrial integrity or secondary effects linked to the immune response.

Biomarkers associated with mitochondrial abnormalities, such as elevated levels of oxidative stress and mitochondrial damage indicators, including F2-isoprostanes and malondialdehyde, alongside reduced antioxidant levels like coenzyme Q10, have been observed (Grossini et al., 2021; Trimarco et al., 2022; Karim et al., 2023; Mikuteit et al., 2023; Mrakic-Sposta et al., 2023).

Consistent with this, blood lactate levels in non-survivors of COVID-19 are significantly higher than in survivors (Carpene et al., 2022). Additionally, in individuals experiencing the long-term effects of COVID-19, there is a tendency for intracellular energy production to favor glycolysis over mitochondrial oxidative phosphorylation. This observation corresponds to potential explanations for post-COVID conditions, such as mitochondrial dysfunction (Nunn et al., 2022).

Genomic studies have also identified expression changes in genes related to mitochondrial function and the cellular response to viral infections in COVID-19 patients (Adamyan et al., 2021; Medini et al., 2021; Wang et al., 2022; Blandova et al., 2024). In recent years, studies have increasingly highlighted the association between circulating mtDNA levels and various pathological conditions, including inflammation, trauma, and viral infections (Ali et al., 2020; Faust et al., 2020; Kim et al., 2021). However, the role of mtDNA during COVID-19 and its long-term consequences remains controversial and requires further investigation (Scozzi et al., 2021; Edinger et al., 2022; Faizan et al., 2022; Shoraka et al., 2023).

Long-term burden on the cardiovascular system is one of the main sequelae of COVID-19 (Lim et al., 2024). Analysis of myocardial tissues from heart failure patients revealed that mitochondrial metabolic disorders and extensive immune inflammation are the most prominent shared features of COVID-19 and cardiovascular diseases. A total of 315 upregulated and 78 downregulated differentially expressed genes were identified. These findings indicate that mitochondrial dysfunction, metabolic dysregulation, and cytokinesis failure play crucial roles in the progression of both COVID-19 and cardiovascular diseases. Additionally, the analysis suggested that SARS-CoV-2 infection impacts pathways associated with the heart, including diabetic cardiomyopathy and adrenergic signaling in cardiomyocytes (Dai et al., 2023).

Aging is linked to a gradual decline in mitochondrial function (Amorim et al., 2022). However, studies in patients revealed that age as an independent risk factor for developing long COVID. Some studies consider age, female gender, higher body mass, and smoking habits as risk factors (Tsampasian et al., 2023; Donald et al., 2024). Conversely, a comprehensive systematic review concluded that the evidence did not support an association between advancing age and long COVID-19, but did support that female sex is a risk factor for long COVID-19 (Notarte et al., 2022).

**FIGURE 1:**
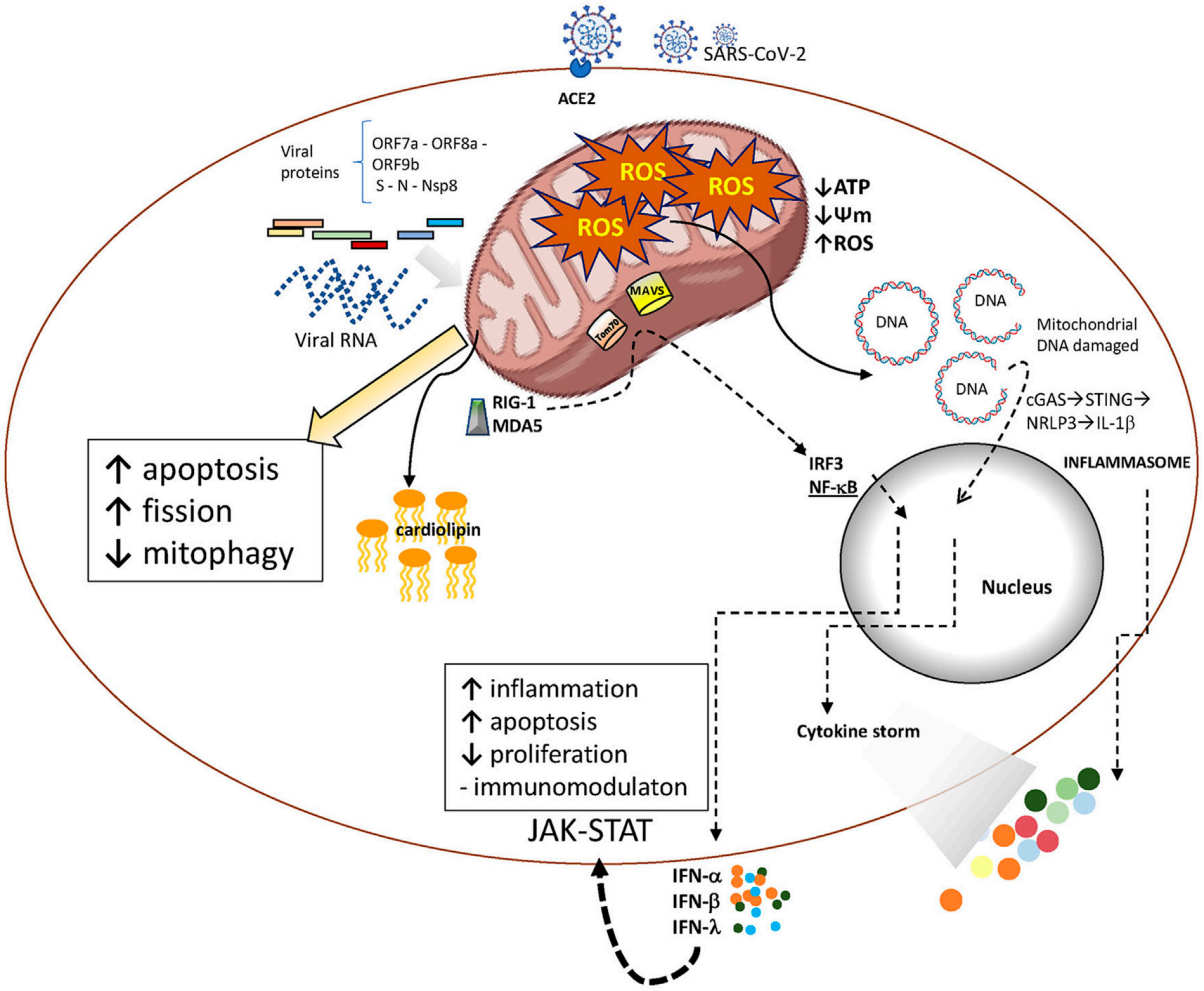
SARS-CoV-2 infection and aging mitochondria. After entering the host cell, SARS-CoV-2 releases its RNA genome, which is subsequently translated into structural and non-structural proteins. Both, viral RNA and proteins act as PAMPS able to trigger innate immune activation pathways that promote cytokines release. Viral proteins may also interact with mitochondrial components, resulting in altered mitochondrial function. These interactions are crucial for the virus to evade mitochondria-mediated innate immune responses and establish infection.

Viral proteins appear to have a direct role in disrupting mitochondrial function by binding to mitochondrial complexes, causing immune cells to overreact. SARS-CoV-2 proteins ORF7a, ORF8a, and ORF9b are known to localize in the mitochondria, where they can inhibit RIG-I and MAVS-dependent interferon signaling (Saxena et al., 2023). This inhibition enhances viral replication, impairs mitochondrial function, and contributes to chronic inflammation (Hartsell et al., 2022). The nucleocapsid (N) protein of SARS-CoV-2, a structural protein essential for viral replication and assembly, has a role in ROS production that has been recently reported (Yu et al., 2023). ROS is also induced by the non-structural protein of SARS-CoV-2, Nsp8. This protein is present in mitochondria and induces mitophagy by reducing mitochondrial membrane potential (Zong et al., 2023). Mitochondrial dysfunction is also induced by the spike (S) protein and is consistent with the differential metabolomic profiles observed in patients with acute respiratory distress syndrome caused by COVID-19 (Figure 1) (Yeung-Luk et al., 2023).

Finally, an increasing number of studies have found that the gut bacterial microbiome in COVID-19 patients undergoes significant changes compared to healthy individuals. Specifically, there is a marked reduction in beneficial commensal bacteria, which are replaced by opportunistic pathogens. Clinical studies indicate a complex interplay between SARS-CoV-2 infection, gut microbiota dysbiosis, mitochondrial dysfunction, and systemic inflammation. This creates a vicious cycle that links “leaky gut” conditions to a disrupted electron transport chain (ETC.), highlighting a quantum leap in molecular and cellular processes (Liu et al., 2022; Vestad et al., 2022).

Overall, while the precise mechanisms linking mitochondrial dysfunction and long COVID remain to be fully elucidated, emerging evidence suggests a link between mitochondrial dysfunction and long COVID symptoms, including fatigue, oxidative stress, immune dysregulation, and viral persistence. Understanding this connection could lead to new therapies targeting mitochondrial function, enhancing biogenesis, promoting mitophagy, reducing ROS with antioxidants, modulating dynamics, boosting ATP production, supporting DNA repair, and reducing inflammation to alleviate symptoms and improve patient outcomes. Consequently, there are research groups actively exploring the possibility of testing such interventions in the laboratory (NCT05373043, NCT05703074, NCT05601180; Ajaz et al., 2021; Barletta et al., 2023; Hansen et al., 2023; Kow et al., 2023).

## Possible role of stressed adipocytes in mitochondria dysfunction and long COVID

The process of aging is linked to the gradual accumulation of visceral white adipose tissue–WAT - (Kyle et al., 2001; Global et al., 2016). Obesity stands out as a primary risk factor for COVID-19 disease (Nour and Altintas, 2023). This association has been linked to a proinflammatory profile and metabolic dysregulation, resulting in severe symptoms and complications such as hypercoagulopathy (Gao, 2020; Han et al., 2021).

Mitochondrial dysfunction is connected to lipid metabolism disorders and increased activity of ACE2 (Zhao et al., 2022). Obesity triggers mitochondrial fragmentation and dysfunction in white adipocytes through the activation of the small GTPase RalA (Xia et al., 2024).

In terms of mitochondrial activity, the presence of excess lipids in obesity leads to the dysregulation of the immune response. Elevated levels of fatty acids hinder the activity of CD4^+^ T-cells, a phenomenon associated with the malfunction of autophagosome formation and mitochondrial degradation (Wen et al., 2022). This disruption impedes mitochondrial turnover, giving rise to increased mtROS and a reduction in ATP levels, both indicative of mitochondrial dysfunction (Kajihara et al., 2017). Additional studies have demonstrated that lipid alterations and high-fat loading interfere with the autophagic process, contributing to the accumulation of damaged mitochondria (Xie et al., 2020).

A growing body of research suggests that adipocytes release extracellular vesicles (EVs) containing a diverse range of load, including lipids, proteins, nucleic acids, and even organelles like mitochondria (Crewe et al., 2018; Crewe et al., 2021). These EVs facilitate communication between cells within WAT and can also have systemic effects on organs located at a distance.

Studies have demonstrated that adipocytes are the primary source of circulating small extracellular vesicle (sEV)-associated miRNAs in mice (Thomou et al., 2017). While the specific cell type responsible for the majority of sEVs in circulation in humans remains uncertain, there is a suggestion that adipocyte-derived sEVs may play a role in whole-body metabolic signaling, potentially at a level that has not been fully recognized (Thomou et al., 2017). However, there is a proposition that EVs derived from adipocytes may significantly contribute to metabolic signaling throughout the entire body, a role that may not have been fully acknowledged to date (Crewe et al., 2021). *In vitro* studies revealed that adipocytes release sEVs containing damaged mitochondria in response to energetic stress, as observed in chronic obesity. The mitochondria associated with these sEVs trigger temporary mitochondrial oxidative stress in cardiac tissue, prompting an antioxidant response. Consequently, adipocyte-derived sEVs play a role in preconditioning the heart, providing protection against ischemia/reperfusion injury. Therefore, these seemingly harmful adipocyte-derived sEVs may offer a physiological pathway for robust cardio-protection against the inevitable lipotoxic or ischemic stresses induced by obesity. Nevertheless, akin to all hermetic responses, it is probable that there exists a threshold for ROS production or a duration of ROS exposure. Beyond this point, instead of providing protection, it may promote damage. Additional research is necessary to discern whether there is a specific stage in the development of obesity where adipocyte-derived sEVs indeed shift from promoting protective effects to inducing damage. Further studies are necessary to establish the link between SARS-CoV-2-mitochondria and obesity.

Can the influenza virus help us understand mitochondrial alterations caused by SARS-CoV-2, long-COVID development, and accelerated aging?

Our understanding indicates that influenza virus and SARS-CoV-2 infections can suppress the host type I IFN responses to evade the immune system. The influenza virus uses non-structural protein 1 (NS1) (Garcia-Sastre, 2001), which, through its interaction with E3 ubiquitin ligase TRIM25, can inhibit IFN-β production by preventing pattern recognition receptors (PRP) activation such as the RNA helicase RIG-I (Gack et al., 2009). For its part, SARS-CoV-2 employs the membrane (M) protein and Orf9b to disrupt IFN signaling. The SARS-CoV-2 M protein can interact with other PRP, the helicase MDA5, interfering with its signaling cascade (TRAF3, IKKε, and TBK1), by inducing degradation of pivotal kinases (TBK1) by ubiquitination. Orf9b interacts with the mitochondrial outer membrane protein TOM70, a 70-kDa membrane-anchored adapter involved in preprotein import into mitochondria, endoplasmic reticulum (ER)-mitochondria contacts, and activation of the antiviral signaling cascade (Gao et al., 2021; Han et al., 2021).

Both viruses interact and inhibit MAVS (mitochondrial antiviral-signaling protein) downstream of RIG-1 and MDA-5, suppressing the immune response mediated by type I IFN. For this goal, the PB1-F2 protein -neo-synthetized during influenza virus replication- (Varga et al., 2012) and the structural M protein of SARS-CoV-2 interact with MAVS (Sui et al., 2021).

Regarding mitochondrial dynamics, SARS-CoV-2 infection appears to increase mitochondrial fusion, resulting in elongation that prevents apoptosis and creates an intracellular environment conducive to virus propagation in infected cells (Shin et al., 2024). Similarly, the influenza virus induces mitochondria hyper-elongation by fission-associated protein DRP1 relocalization to the cytosol, enhancing a pro-fusion status (Pila-Castellanos et al., 2021) and the mitophagy through the NOD2 receptor and the cytosolic protein kinase RIPK2 (Lupfer et al., 2013). The influenza virus PB1-F2 protein disrupts mitochondrial membrane potential and downregulates immune responses, leading to decreased IL-18 secretion and inflammasome activation by affecting NLRP3 activation (Gibbs et al., 2003). Conversely, SARS-CoV-2 induces exaggerated inflammatory responses, recruiting monocytes and macrophages that release cytokines, leading to severe lung damage and chronic pathology. Additionally, mitochondrial dysfunction produces oxidative stress, leading to platelet dysfunction and activation of coagulation pathways (Georgieva et al., 2023).

These differences in how each virus interacts with mitochondria and the resulting immune response could partly explain why hospitalized COVID-19 patients are at a higher risk of long-term complications than influenza survivors (Liu et al., 2023).

Understanding the complex interactions between viruses and host cell mitochondria is crucial for developing targeted antiviral therapies and managing viral infections. Both viruses affect mitochondrial function and, consequently, the immune response. Although the mechanism underlying long-term complications, such as long-COVID, is not fully elucidated, it is likely related to mitochondrial dysfunction. Therefore, we could speculate that antioxidant therapies or the transfer of functional mitochondria might rescue the host cell from death.

## Conclusion

The diverse clinical presentations of COVID-19, ranging from asymptomatic cases to severe respiratory issues and fatalities, present significant challenges in global pandemic management. Severe outcomes are often linked to an intensification of inflammatory processes, causing systemic problems such as oxidative stress, disruptions in iron balance, increased blood clotting, and the formation of thrombi.

The severity of COVID-19 is elevated in elderly individuals with obesity, conditions closely associated with mitochondrial dysfunction. Dysfunctional mitochondria compromise the immune system’s antiviral response, influencing the release of type I and Type III interferons and exacerbating viral pathogenesis. The interaction between the SARS-CoV-2 virus and mitochondria modulates the immune response, potentially impacting disease progression.

Mitochondrial dysfunction induced by obesity, especially in adipocytes, may contribute to metabolic dysregulation and proinflammatory profiles.

The persistent symptoms observed in long COVID patients may be linked to mitochondrial dysfunction, reactive oxygen species generation, and a shift to glycolysis.

The review underscores the necessity for further research into the role of mitochondria in elderly COVID-19 patients, particularly those with age-dependent fat accumulation. The ultimate goal is to develop strategies that can mitigate the long-term consequences of the disease.
